# Evaluation of the HIV-1 Polymerase Gene Sequence Diversity for Prediction of Recent HIV-1 Infections Using Shannon Entropy Analysis

**DOI:** 10.3390/v14071587

**Published:** 2022-07-21

**Authors:** Paballo Nkone, Shayne Loubser, Thomas C. Quinn, Andrew D. Redd, Oliver Laeyendecker, Caroline T. Tiemessen, Simnikiwe H. Mayaphi

**Affiliations:** 1 Department of Medical Virology, University of Pretoria, Tshwane 0031, South Africa; u16124155@tuks.co.za; 2 National Institute for Communicable Diseases and Faculty of Health Sciences, University of the Witwatersrand, Johannesburg 2000, South Africa; shaynel@nicd.ac.za (S.L.); carolinet@nicd.ac.za (C.T.T.); 3 Division of Intramural Research, National Institute of Allergy and Infectious Diseases, National Institutes of Health, Bethesda, MD 20892, USA; tquinn2@jhmi.edu (T.C.Q.); aredd2@jhmi.edu (A.D.R.); olaeyen1@jhmi.edu (O.L.); 4 Department of Medicine, Johns Hopkins University, Baltimore, MD 21231, USA; 5 National Health Laboratory Service-Tshwane Academic Division (NHLS-TAD), Tshwane 0031, South Africa

**Keywords:** recent HIV-1 infection, chronic HIV-1 infection, Shannon entropy, sequence diversity, false recency rate, HIV-1 polymerase, sanger sequencing

## Abstract

HIV-1 incidence is an important parameter for assessing the impact of HIV-1 interventions. The aim of this study was to evaluate HIV-1 polymerase (*pol*) gene sequence diversity for the prediction of recent HIV-1 infections. Complete *pol* Sanger sequences obtained from 45 participants confirmed to have recent or chronic HIV-1 infection were used. Shannon entropy was calculated for amino acid (aa) sequences for the entire *pol* and for sliding windows consisting of 50 aa each. Entropy scores for the complete HIV-1 *pol* were significantly higher in chronic compared to recent HIV-1 infections (*p* < 0.0001) and the same pattern was observed for some sliding windows (*p*-values ranging from 0.011 to <0.001), leading to the identification of some aa mutations that could discriminate between recent and chronic infection. Different aa mutation groups were assessed for predicting recent infection and their performance ranged from 64.3% to 100% but had a high false recency rate (FRR), which was decreased to 19.4% when another amino acid mutation (M456) was included in the analysis. The *pol*-based molecular method identified in this study would not be ideal for use on its own due to high FRR; however, this method could be considered for complementing existing serological assays to further reduce FRR.

## 1. Introduction

Over the years various HIV-1 intervention programs have been implemented, which have led to a gradual decline in new HIV-1 infections and AIDS-related deaths [1,2]. These interventions include, but are not limited to, affordable HIV-1 testing at local health care facilities, scale up of antiretroviral therapy (ART) to both adults and young children, and treatment monitoring [3,4,5].

The 2019 coronavirus disease (COVID-19) pandemic and lockdown restrictions disrupted HIV-1 management and prevention programs, especially in low and middle-income countries like South Africa (SA) [6,7]. Patients who were newly infected with HIV-1 had a delayed diagnosis and treatment initiation as local clinics were closed, and those already on treatment had longer waiting times to finally gain access to nearby health care centres [7,8].

HIV-1 incidence is the measure of the rate of new HIV-1 infections in the population over a defined period, and it is an important parameter for assessing the impact of HIV-1 interventions [6,9]. Currently used approaches for determining HIV-1 incidence include longitudinal studies, inference from prevalence measures, and cross-sectional studies [9]. Cross-sectional studies use once-off sampling and focus on measuring the incidence rate using biological markers that can distinguish early/recent from chronic HIV-1 infection [10,11]. Recent HIV-1 infection is defined as the period from acquiring the virus until the viral set-point is achieved, which takes approximately six months. Chronic HIV-1 infection is the stage that begins after the set-point is established and ends when the AIDS stage starts [12,13].

HIV-1 avidity assays are often used for diagnosing recent HIV-1 infections in cross-sectional studies [10,14,15]. Multi-assay algorithms (MAAs) have been incorporated into cross-sectional studies and are a promising alternative to identifying recent infections [14,16]. MAAs usually consist of a limiting antigen avidity enzyme immunoassay (LAg-Avidity assay) with other assays, such as viral load (VL) or the cluster of differentiation 4 (CD4) count [9,14]. The LAg-Avidity assay measures the avidity of immunoglobulin G (IgG) antibodies against HIV-1 in serum or plasma samples. People with recent infection have a low IgG avidity as compared to those with long-term or chronic infection, therefore this assay is used to discriminate between recent and chronic infections [17]. The drawback of this assay is that it can misclassify chronic HIV-1 infections as recent (false recency) in some cohorts and that various assay cutoffs of normalised optical density units have to be examined for a population [9,14]. This highlights a need for developing alternative assays for the detection of incident HIV-1 infections. The aim of this study was to evaluate the HIV-1 *pol* sequence diversity for the prediction of recent HIV-1 infections using Shannon entropy analysis.

## 2. Materials and Methods

### 2.1. Study Population

This was a retrospective study that used stored samples collected from individuals in the Tshwane district of South Africa. Participants enrolled in this study had a confirmed diagnosis of recent or chronic HIV-1 infection and were ART naïve. They were identified in a study that screened for recent HIV-1 infection in individuals who had a negative rapid test at the point-of-care facilities, and diagnosis was confirmed through HIV nucleic acid amplification test (NAAT), antibody (on 3rd generation enzyme-linked immunoassay), p24 antigen, Western blot and LAg avidity testing. Participants were classified as having recent HIV infection if they had detectable HIV RNA with negative/positive p24 antigen or HIV-specific antibodies, and low LAg avidity assay normalised optical density (OD-n) values (<1.5) if antibodies were detected. Participants who had detectable HIV RNA with negative/positive p24 antigen, and detectable HIV-specific antibodies with high avidity (>1.5 OD-n) were classified as having a chronic HIV infection. [18]. Plasma samples obtained at one-time point were used in this study, thus reflecting a cross-sectional analysis of a *pol*-based molecular strategy to predict recent HIV-1 recent infections.

### 2.2. Nucleic Acid Extraction and Amplification of HIV-1 pol

Total nucleic acids were manually extracted from plasma samples using the QIAamp UltraSens Virus Kit (Qiagen, Hilden, Germany). The complete HIV-1 *pol* gene was amplified in all samples using an in-house nested PCR method, employing the SuperScript™ III One-Step RT-PCR System with Platinum™ *Taq* High Fidelity DNA Polymerase (Invitrogen, Carlsbad, CA, USA). The full nested PCR protocol and set of primers used were outlined in a previous study [19].

### 2.3. Sanger Sequencing and Sequence Analysis

Complete HIV-1 *pol* Sanger sequences obtained from a previous study [20] were used for analysis. Sanger sequencing was performed on PCR amplicons in five overlapping regions covering the entire *pol* open reading frame (ORF) (Inqaba Biotechnical Industries, Tshwane, South Africa). Sequences were analysed and edited as mentioned in our previous publication [19]. In this study, only subtype C sequences were used for analysis.

### 2.4. Shannon Entropy Analysis for Measuring Diversity

Entropy measures the variability within a site and assigns a high score to highly variable sites and a lower score to less variable sites. Complete HIV-1 *pol* Sanger sequences from our study were grouped into recent HIV-1 or chronic HIV-1 sequences and aligned to an HIV-1 subtype C reference (AY162225). The aligned amino acid (aa) sequences were submitted to the Los Alamos National Laboratory (LANL) website (https://www.hiv.lanl.gov/content/sequence/ENTROPY/entropy_one.html (accessed on: 9 February 2022)) to calculate the Shannon entropy for each amino acid site. Further analysis focused on identifying nonsynonymous amino acid mutations that can be used to differentiate recent from chronic HIV-1 infections. The ratio of nonsynonymous to synonymous (dN-dS) nucleotide mutations was not assessed in this study as it has already been performed for the same dataset in our previous study [19].

Initially, Shannon entropy scores were computed for the complete *pol* gene (913 aa) followed by a comparison of the median scores between recent and chronic HIV-1 infections. This was then followed by screening for sequence diversity (entropy scores) in different regions of *pol* using the sliding window feature. Windows of different aa lengths were explored at first, these included window sizes of 50 aa, 100 aa, 150 aa and 200 aa lengths. A window size of 50 aa was chosen for screening as it enabled coverage of almost the entire *pol* aa sequence length (except for the last 13 aa), while also keeping the sequences shorter for further analysis. The median entropy scores between recent and chronic HIV-1 infections were compared for each window so as to assess specific regions that can discriminate between recent and chronic HIV-1 infection stages.

Amino acid sequences that corresponded with sliding windows whose median scores were significantly different between recent and chronic HIV-1 infection were further analysed in BioEdit 7.2.5. In each window, amino acids that showed a different pattern or frequency between the two stages of infection were observed and documented. We then compared our findings to reference sequences from LANL/GenBank, which were obtained from cohorts with recent or chronic HIV-1 stages (Supplementary Table S1).

### 2.5. Reference Sequence Data Sources

Reference sequences were mined from the LANL HIV sequences database/GenBank (https://www.hiv.lanl.gov/components/sequence/HIV/search/search.html (accessed on: 9 February 2022)). HIV-1 subtype C sequences were obtained from past studies in which the participants were clearly identified to have recent HIV-1 (defined as an infection duration of <6 months regardless of the Fiebig staging) or chronic HIV-1 (defined as infection duration of >6 months) and all sequences were from ART-naïve participants. Ninety six recent HIV-1 infection reference sequences and 136 chronic infection reference sequences were obtained from past studies (Supplementary Table S1). Non-subtype C references were also evaluated to assess if this study’s findings were applicable to them.

### 2.6. Statistical Analysis

Descriptive statistics were used to present the median values and the interquartile range (IQR) for age, mean values (range) for CD4 count and HIV-1 VL and the proportion of participants with recent or chronic HIV-1 infection. Numerical variables were assessed for normal distribution, and the absolute HIV-1 VL values were log-transformed before analysis. A two-sample t-test was used to compare the mean CD4 count and HIV-1 VL log_10_ values between recent and chronic HIV-1 stages. The trends of existence of some mutations between recent and chronic HIV-1 stages were documented, and the Fisher’s exact test was used to assess if there was an association between the distribution of these mutations and HIV-1 disease stage. Shannon entropy data for the complete *pol* and for each sliding window was imported into GraphPad Prism 9.2.0 (https://www.graphpad.com/scientific-software/prism/ (accessed on: 24 February 2022)) and median entropy scores of recent and chronic infections were compared using an unpaired *t*-test. A *p*-value of ≤0.05 was considered significant. Scatterplots were created to show the median difference in entropy scores.

## 3. Results

### 3.1. Demographics

This study enrolled a total of 45 HIV-1 infected participants. Their median age was 28 years (IQR: 24–32 years) and majority (93.3%) were females. There were 14 participants (31.1%) with recent HIV-1 infection and 31 (68.9%) with chronic HIV-1 infection. The mean HIV-1 VL was significantly higher in participants with recent infection (5.21 log_10_ copies/mL [3.23–8.52 log_10_ copies/mL]) compared to those with chronic infection (4.22 log_10_ copies/mL ([2.59–5.32 log_10_ copies/mL]), *p* = 0.01. The mean CD4 count was also significantly higher in participants with recent HIV-1 (555 cells/μL [215–964 cells/μL]) compared to those with chronic HIV-1 (362 cells/μL [72–675 cells/μL]), *p* = 0.02 (Table 1).

### 3.2. Sequencing and Complete HIV-1 pol Shannon Entropy

**Figure 1:**
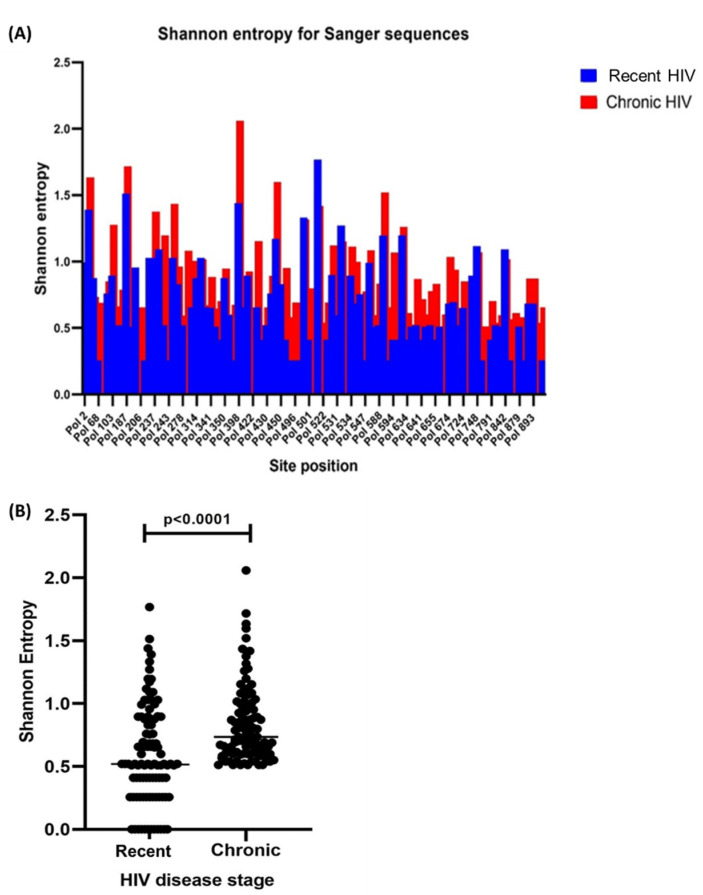
Shannon entropy score analysis of the complete HIV polymerase gene amino acid sequences obtained during recent and chronic HIV-1 disease stages. (**A**) Higher diversity (entropy) was observed during chronic HIV-1 infection with site-by-site entropy analysis. (**B**) A scatterplot indicating a significant difference in the median entropy scores between the two stages of infection. Graphs were plotted using GraphPad Prism. HIV = Human immunodeficiency virus.

All sequences used for analysis were from individuals infected with HIV-1 subtype C. Shannon entropy analysis performed for the complete HIV-1 *pol* gene showed that there was generally a higher diversity observed in chronic HIV-1 infection compared to recent HIV-1 infection. There was a statistically significant difference between the median entropy scores obtained from recent and chronic HIV-1 infection stages (*p* < 0.0001) (Figure 1).

### 3.3. Amino Acid Diversity within HIV-1 pol Sliding Windows

**Figure 2:**
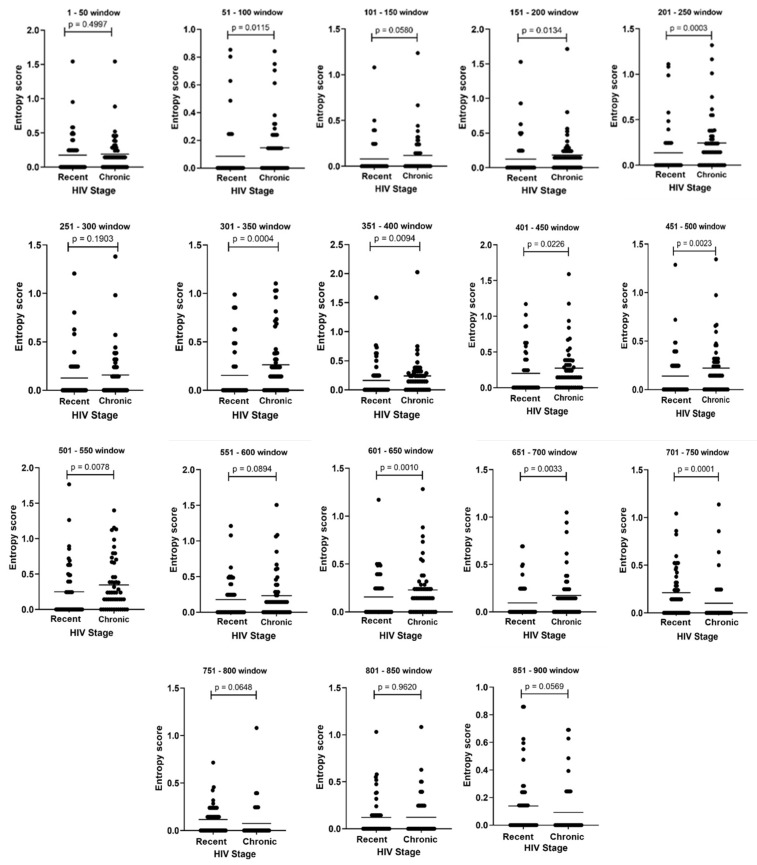
Scatterplots of sliding windows (containing 50 amino acids each) used to screen for informative areas that can differentiate recent from chronic infection. The following windows had significantly higher median entropy scores between recent and chronic HIV disease stages: 51–100, 151–200, 201–250, 301–350, 351–400, 401–450, 451–500, 501–550, 601–650, 651–700 and 701–750. Scatterplots were created using GraphPad Prism. HIV = Human immunodeficiency virus.

Through the sliding window analysis, 11 out of 18 windows were found to have significantly higher median entropy scores with chronic HIV-1 sequences compared to recent HIV-1 sequences, with *p* values ranging from 0.011 to <0.001 (Figure 2).

Further amino acid sequence analysis was performed in the windows that showed statistical significance between recent and chronic HIV-1 infection stages, and only a few windows had notable amino acid mutations whose distribution or frequency was different between the recent and chronic HIV-1 stages. There were 13 amino acid mutations that were identified to have a different distribution between recent and chronic HIV infection stages, and some of these could be found in the same sliding window. Interestingly, almost all of these amino acid mutations had a higher frequency in the chronic HIV-1 stage; however, none showed statistical significance (Table 1 and Supplementary Figure S1). All these amino acid mutations were located in the recognized cytotoxic T-lymphocyte (CTL) epitopes within the reverse transcriptase (RT) and integrase (IN) regions of HIV-1 *pol* (Table 2). There was more variability observed with nucleotide analysis of the codons coding for the identified amino acid mutations (Supplementary Table S2).

**Figure 3:**
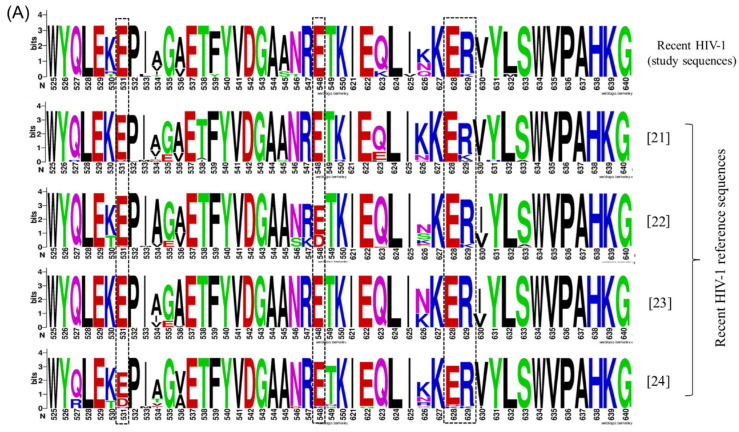

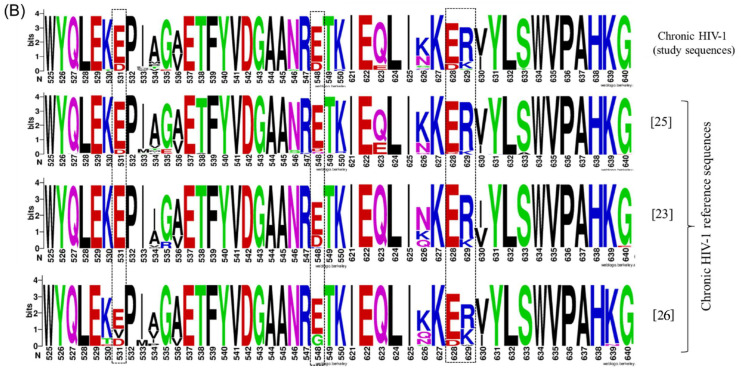
Amino acid mutations (E531, E548, E628 and R629) identified in study sequences to be highly conserved during recent HIV-1 stage compared to chronic HIV stage. (**A**) Comparison of these amino acid mutations with subtype C reference sequences obtained during recent HIV-1 infection [21,22,23,24]. (**B**) Comparison of these amino acid mutations with subtype C reference sequences obtained during chronic HIV-1 infection [23,25,26]. Image created in WebLogo 3.7.4. HIV = human immunodeficiency virus.

There were interesting similarities between the findings of this study and most subtype C reference sequences regarding the distribution of identified amino acid mutations that can discriminate between recent and chronic HIV-1 stages. The analysis of these reference sequences also showed that the chronic HIV-1 stage has higher sequence diversity and that most highly informative amino acid sites identified in study sequences were also more conserved during recent HIV-1 infection (Table 3 and Figure 3). Non-subtype C reference sequences were also evaluated for comparison, but most of these had a different distribution of amino acid mutations compared to that observed in study sequences (Supplementary Figure S2).

### 3.4. A pol-Based Molecular Strategy to Detect Recent HIV-1 Infection

This study identified amino acid mutations that could discriminate between recent and chronic infection (Table 1 and Supplementary Figure S1). However, these amino acids were not always present or absent in each stage of infection for all participants, thus making it difficult to use an individual amino acid for predicting recent HIV-1 infection. This led to the assessment of a combination effect of these amino acids for predicting recent HIV-1 infection. Different groups of amino acid mutations designated IT, ER and EL (named based on the first and last amino acid in each group) were evaluated. The performance of these groups for predicting recent HIV-1 infection ranged from 64.3–100%. However, all these combinations had a high false recency rate (FRR) ranging from 35.5–48.4%. The ER group showed a performance of 100% for predicting recent infection as it had highly conserved amino acid sites during recent infection compared to chronic HIV-1 infection (Table 1 and Figure 3). When the M456 amino acid mutation (within the IT group) was added to ER and EL for further analysis, it led to a reduction in FRR to 29.0% and 19.4%, respectively. However, the addition of M456 to these groups also reduced their performance (Table 4). In this study, a strategy of including VL and/or CD4 count with the existing amino acid combinations for assessing recent HIV-1 was also analysed but it did not show improved results as some participants with chronic infection had high CD4 count and VL (Table 1).

## 4. Discussion

This study looked at evaluating a molecular-based strategy for identifying recent infection through Shannon entropy analysis of HIV-1 *pol* sequences. The characteristics of study participants were suitable for such a purpose as these were ART-naïve participants and were clearly defined to have either recent or chronic HIV-1 infection [18], and therefore the diversity in their sequences is more likely due to natural selection pressure. Such criteria are similar to that of past studies that evaluated the performance of serological assays for diagnosis of recent HIV-1 infection [27,28]. The advantage of this study is that it evaluated the sequence diversity in the entire *pol* gene and that reference sequences were used to verify the study findings.

The significantly higher diversity observed with chronic sequences has been previously reported in other studies, indicating that as the duration of infection increases, so does sequence diversity within the virus [25,29,30]. Such diversity in sequences from ART-naïve participants can be attributed to CTL responses as all the amino acid mutations were mapped to recognized CTL epitopes. Our previous work showed that HIV-1 *pol* evolves with time due to continuous immune selection pressure [19]. Another factor that may contribute to higher diversity in *pol* sequences during chronic is that *pol* contains more subdominant CTL epitopes that tend to be recognized by CTL immune responses later in the course of infection [31,32].

This study identified a number of informative regions and amino acid mutations within the HIV-1 *pol* gene that can discriminate between recent and chronic HIV-1 infection stages (Table 1 and Figure 2). This highlights that the Shannon entropy analysis of sliding windows coupled with the further analysis of these windows on BioEdit is a good strategy of screening for these informative regions. Interestingly, these regions were easily identified through the analysis of amino acid sequences as opposed to nucleotide sequences. This is mainly due to synonymous nucleotide mutations, which make the diversity seem higher when analysing nucleotide sequences. Wu et al. previously reported that within-host diversity is variable across different regions of the virus to an extent that some regions may be more predictive of recent infection than others [33]. The observation made from the subtype C reference sequences regarding a similar pattern of amino acid mutations that can differentiate between the two stages of HIV-1 infection (Table 3 and Figure 3) strengthens the findings of our study [21,22,23,24,26]. A Malawi-based study that had sequences obtained in both recent and chronic HIV-1 stages also showed a similar pattern of amino acid mutations, except for a few amino acid sites that were still conserved even within chronic sequences [23]. Generally, the ER group was also quite conserved among reference sequences obtained during recent infection, except for some variability in a few sites, which could be explained by PCR or sequencing errors or different human leukocyte antigen (HLA) profiles of the study participants [21,22,23,24,26]. The difference in patterns of mutations that were seen with non-C subtypes can be explained by the differences in consensus sequences among different subtypes within HIV-1 group M, in which mutations found in subtype C could be part of a consensus in another subtype [34,35]. This highlights that there could be subtype variations for a molecular-based strategy for the detection of recent HIV-1 infection.

The combination of amino acid mutations for prediction of recent HIV-1 infections showed promising results but all the groups of mutations assessed had high FRR. Interestingly, this high FRR is comparable to what was initially observed with avidity assays such as BED-capture and LAg-avidity in their early stages of development [14,36]. In some studies that assessed the performance of these avidity assays, the FRR ranged from 12% to 45% for LAg-avidity and was as high as 17% for BED-capture [14,37]. However, the FRR of these avidity assays has been markedly improved over time [37,38,39]. The high FRR observed in this study could be due to HIV-1 *pol* being a highly conserved region amongst HIV-1 genes [34], thus its diversity may not be as highly discriminatory between recent and chronic HIV-1 stages [34]. Future studies should consider Shannon entropy analysis of the group-specific antigen (*gag*) and envelope (*env*) sequences as these genes are more variable compared to the *pol* gene, thus could improve discrimination between recent and chronic HIV infections [34].

Past studies have looked at studying sequence diversity to distinguish recent from chronic infection and have mostly used sequence viewing programs like BioEdit with mathematical models to analyse the data and predict recency [29,30,40]. These studies have proven that sequence mutations have sufficient discriminatory power to predict recent from chronic infection and have shown great concordance when compared to the performance of some serological assays [29,30]. Molecular methods for predicting recent HIV-1 infection have been proposed in these studies but none provided a clear strategy for identifying regions that are highly informative for detecting recent infection [30,40,41]. Only a few studies have shown that Shannon entropy is a useful tool for predicting recent infection, but these studies mainly used longitudinal sequence data, thus it might be difficult to use their findings to inform a strategy for use at a population level [33,41]. Our study has an advantage in that it employed a cross-sectional analysis, which would make it easier to implement its findings if they were to be adopted for routine diagnostic use at a population level.

The limitations of this study include a small sample size, particularly for participants with recent HIV-1 infection, and that it mainly focused on HIV-1 subtype C. However, we tried to address this limitation by extending the analysis to 232 reference sequences obtained from patients with either recent or chronic HIV-1 infection. Interestingly, analysis of reference sequences revealed similar findings as those observed from the study sequences. The performance of the identified groups of amino acid mutations could not be assessed at different durations of infection owing to fewer participants with recent HIV-1 infection. The duration of the chronic infection in our study is not known, which limited our analysis for different durations of infection beyond 6 months. Very few reference studies had both recent and chronic sequences to enable easier comparison with this study.

## 5. Conclusions

Our data highlight a method that can be used to identify highly informative regions within an HIV-1 gene, which can be used to discriminate between recent and chronic infection. The *pol*-based molecular method identified in this study would not be ideal for use on its own due to the high FRR. However, this method could be used to complement existing serological assays for the prediction of recent infection in order to further reduce the FRR observed with serology assays. The advantage of a *pol*-based molecular strategy would be that the HIV-1 *pol* gene is sequenced in some countries for antiretroviral (ARV) drug resistance testing before patients are initiated on ART. Further research is needed for the development of a molecular-based method for the detection of recent HIV-1 infections.

## Figures and Tables

**Table 1:** Demographics and summary of the amino acid mutations found to have a different distribution between recent and chronic infection sequences.

							IT			ER				EL				
Pt ID	HIV Stage	Sex	Age (years)	HIV VL (copies/mL)	CD4 Count (cells/uL)	I234	I241	M456	T499	E531	E548	E628	R629	E670	E684	I690	L704	L733
261	R	M	33	$8.4\;\times \;10^{7}$	386	-	-	-	-	-	-	-	-	-	-	-	-	-
2504	R	F	24	$3.7\;\times \;10^{4}$	287	X	-	X	-	-	-	-	-	-	-	X	-	X
5041	R	M	23	$2.2\;\times \;10^{7}$	n/a	-	-	-	-	-	-	-	-	-	-	-	-	
6512	R	F	23	$1.7\;\times \;10^{3}$	215	-	-	-	-	-	-	-	-	-	-	-	-	-
6727	R	F	28	$4.8\;\times \;10^{3}$	706	X	-	-	-	-	-	-	-	-	-	-	-	-
6638	R	F	28	$1.9\;\times \;10^{5}$	457	X	-	-	-	-	-	-	-	-	-	-	-	-
6582	R	F	24	$6.2\;\times \;10^{3}$	818	-	-	-	-	-	-	-	-	-	-	-	-	-
9498	R	F	34	$5.0\;\times \;10^{5}$	964	-	-	-	X	-	-	-	-	-	X	-	-	-
9049	R	F	20	$1.6\;\times \;10^{4}$	n/a	-	-	-	-	-	-	-	-	-	-	-	-	-
8575	R	F	27	$9.3\;\times \;10^{4}$	668	-	-	X	X	-	-	-	-	-	X	-	-	-
8047	R	M	31	$1.2\;\times \;10^{6}$	n/a	X	-	-	-	-	-	-	-	-	-	X	-	-
7084	R	F	28	$3.3\;\times \;10^{8}$	411	-	-	X	-	-	-	-	-	X	-	-	-	-
6743	R	F	26	$2.7\;\times \;10^{4}$	638	-	-	-	-	-	-	-	-	-	-	-	-	X
6737	R	F	24	$2.2\;\times \;10^{3}$	n/a	X	-	-	-	-	-	-	-	-	-	-	-	X
639	C	F	30	$6.5\;\times \;10^{3}$	392	X	-	-	-	X	-	-	-	-	-	-	X	-
843	C	F	21	$2.9\;\times \;10^{4}$	348	-	X	X	-	-	-	-	X	-	-	-	-	-
1121	C	F	27	$8.0\;\times \;10^{4}$	127	-	-	-	-	-	X	-	-	X	-	-	-	-
1213	C	F	36	$3.2\;\times \;10^{4}$	n/a	-	-	-	-	-	X	-	-	-	-	-	-	-
1475	C	F	32	$4.4\;\times \;10^{4}$	255	-	-	X	X	-	X	X	-	-	-	X	-	-
2340	C	F	22	$1.4\;\times \;10^{4}$	n/a	-	-	-	-	-	-	-	X	-	X	X	-	-
2696	C	F	18	$3.9\;\times \;10^{2}$	n/a	-	-	-	-	-	-	-	-	-	-	-	-	-
3253	C	F	28	$8.9\;\times \;10^{4}$	n/a	-	-	-	-	X	-	-	X	X	X	X	-	-
3387	C	F	37	$7.9\;\times \;10^{4}$	n/a	-	-	X	-	-	X	-	-	-	-	-	-	-
3474	C	F	21	$1.6\;\times \;10^{4}$	675	-	-	X	-	-	-	-	-	X	-	-	-	-
9986	C	F	28	$9.7\;\times \;10^{4}$	160	-	X	X	-	-	-	-	-	-	-	X	-	-
3606	C	F	24	$1.5\;\times \;10^{4}$	529	-	-	-	-	-	-	-	-	-	-	-	X	-
3869	C	F	32	$2.1\;\times \;10^{5}$	n/a	-	-	X	-	-	-	-	-	-	-	X	-	-
3880	C	F	30	$7.5\;\times \;10^{3}$	575	-	-	-	-	-	-	-	-	X	-	-	X	-
3910	C	F	33	$2.8\;\times \;10^{4}$	n/a	-	-	-	-	X	-	X	-	-	-	-	X	-
3912	C	F	27	$3.2\;\times \;10^{4}$	287	-	-	-	-	-	-	-	X	X	-	-	-	-
3920	C	F	20	$6.6\;\times \;10^{4}$	306	-	-	X	-	-	-	-	X	-	-	-	-	-
4351	C	F	35	$2.6\;\times \;10^{3}$	n/a	-	X	X	-	X	-	-	-	-	-	-	-	-
4198	C	F	19	$1.7\;\times \;10^{3}$	n/a	-	-	-	-	-	-	-	-	-	-	-	-	-
5054	C	F	26	$2.7\;\times \;10^{4}$	72	-	-	X	-	-	-	-	-	-	-	-	-	-
6380	C	F	25	$1.1\;\times \;10^{4}$	385	-	X	-	-	-	-	-	-	-	-	-	X	-
6565	C	F	28	$5.6\;\times \;10^{3}$	371	-	-	-	-	-	-	-	-	-	X	-	-	-
6671	C	F	35	$1.4\;\times \;10^{4}$	n/a	-	-	-	X	-	X	-	-	-	-	-	-	-
6649	C	F	32	$2.1\;\times \;10^{4}$	164	-	-	X	X	-	-	-	-	-	-	X	-	-
6640	C	F	37	$3.0\;\times \;10^{3}$	407	-	-	-	-	-	-	X	-	-	-	X	X	-
6596	C	F	31	$3.8\;\times \;10^{3}$	576	X	-	-	-	X	-	-	X	-	-	X	-	-
9915	C	F	30	$1.4\;\times \;10^{4}$	382	-	-	-	X	-	-	-	-	-	X	-	-	-
9895	C	F	31	$4.8\;\times \;10^{3}$	607	-	-	-	-	-	-	-	-	-	-	-	-	-
9854	C	F	20	$1.2\;\times \;10^{4}$	n/a	-	-	X	-	-	-	-	-	X	-	-	X	-
7959	C	F	40	$1.4\;\times \;10^{5}$	343	-	-	X	X	-	-	X	-	X	X	-	-	-
6990	C	F	26	$1.7\;\times \;10^{4}$	269	-	-	-	X	-	-	-	-	-	-	-	-	-

Different groups of amino acid mutations (ER, EL and IT) were named based on the first and last amino acid of the group. The amino acid mutations (I234 and L733) had higher frequency in recent infection but were not used for further analysis as there were only few of these mutations identified during recent HIV stage. There was no statistical significance (*p* > 0.05) for amino acid mutations with high frequency during chronic HIV-1 infection. Pt ID = participant identity; R = recent HIV; C = chronic HIV; F = female; M = male; n/a = not available.

**Table 2:** HIV-1 pol CTL epitopes and location of amino acid mutations with different distribution between recent and chronic infection stages.

Amino Acid Site	CTL Epitope Mapped to	HIV-1 *pol* Position	Subtype Identified for
I241	NETPGIRYQ	RT (137–145)	B
M456	RMRTAHTNDVK	RT (356–366)	B, C
T499	PIQKETWETW	RT (392–401)	B
E531	EPIAGAETFY	RT (432–441)	C
E548	RETKLGKAGY	RT (448–457)	--
E628	IKKEEVYLA	RT (526–534)	B
E629	IKKEEVYLA	RT (526–534)	B
E670	QEEHEKYHNSW	IN (9–19)	B, C
E684	RAMASEFNL	IN (20–28)	--
I690	FNLPPIVAKEI	IN (26–36)	A
L704	VASCDKCQL	IN (37–45)	C

CTL = cytotoxic T-lymphocyte; RT = reverse transcriptase; IN = integrase; -- = HIV-1 subtype for this epitope has not yet been defined. Underlined = mutant amino acids within these epitopes.

**Table 3:** Comparison of the frequency of highly informative amino acid mutations between study sequences and reference sequences.

		IT			ER				EL			
Study Sequences	n	I241	M456	T499	E531	E548	E628	R629	E670	E684	I690	L704
Recent	14	0	3	2	0	0	0	0	1	2	2	0
Chronic	30	4	12	6	5	5	4	6	7	5	8	7
**Recent reference sequences**												
SA [21]	21	2	5	1	1	0	0	4	5	3	5	3
SA [22]	29	2	6	16	1	8	0	2	2	3	8	1
Malawi [23]	25	0	0	0	0	0	0	0	0	15	9	15
India [24]	21	1	3	2	6	1	2	1	3	21	8	2
**Chronic reference sequences**												
SA [25]	75	3	23	21	7	8	5	5	7	12	15	12
Malawi [23]	38	0	6	0	0	9	0	5	9	7	0	10
Botswana [26]	23	7	12	10	11	6	3	14	6	15	19	0

Different groups of amino acid mutations (ER, EL and IT) were named based on the first and last amino acid of the group. Highly informative amino acid mutations are mutations that could discriminate between recent and chronic HIV-1 infections.

**Table 4:** Performance of the different combinations of amino acid mutations for detecting recent HIV-1 infection.

Stage	n	ER	ERM	EL	ELM	IT
<6 months	14	14 (100%)	11 (78.6%)	9 (64.3%)	9 (64.3%)	10 (71.4%)
>6 months	31	15 (48.4%)	9 (29.0%)	11 (35.5%)	6 (19.4%)	15 (48.4%)

Different groups of amino acid mutations (ER, EL and IT) were named based on the first and last amino acid of the group. See Table 1 for the distribution of mutations in recent compared to chronic infection. The M456 mutation was used in combination with other amino acid mutations outside its group (see Table 1), this led to new groups designated ERM and ELM. <6 months = recent infection; >6 months = chronic infection.

## Data Availability

All data generated or analysed during this study are included in this manuscript and its supplementary information files.

## Supplementary Materials

The following supporting information can be downloaded at: https://www.mdpi.com/article/10.3390/v14071587/s1, Supplementary Table S1: List of articles from which HIV-1 subtype C reference sequences were obtained; Supplementary Table S2: Nucleotide differences within amino acid mutations found to have a different distribution between recent and chronic infection sequences; Supplementary Figure S1: Amino acid sequence alignment to show the difference in sequences between recent and chronic HIV-1 infection; Supplementary Figure S2: Comparison of differences in amino acids E628 and R629 in study sequences and non-subtype C reference sequences, for recent and chronic sequences.

## Abbreviations

aa	Amino acid
AIDS	Acquired immunodeficiency syndrome
ART	Antiretroviral therapy
ARV	Antiretroviral
CD4	Cluster of differentiation 4
COVID-19	Coronavirus disease of 2019
CTL	Cytotoxic T-lymphocyte
dN-dS	Nonsynonymous to synonymous ratio
Env (*env*)	Envelope
FRR	False recency rate
Gag (*gag*)	Group-specific antigen
HIV-1	Human immunodeficiency virus type 1
IgG	Immunoglobulin G
IN	Integrase
IQR	Interquartile range
LAg	Limiting antigen avidity
LANL	Los Alamos National Laboratory
MAA	Multi-assay algorithm
NAAT	Nucleic acid amplification test
OD-n	Normalised optical density
ORF	Open reading frame
Pol (*pol*)	Polymerase
RT	Reverse transcriptase
RT-PCR	Reverse transcription polymerase chain reaction
VL	Viral load
