# Supplementary material for: Evaluation of the HIV-1 Polymerase Gene Sequence Diversity for Prediction of Recent HIV-1 Infections Using Shannon Entropy Analysis
Source: Viruses. 2022 Jul 21;14(7):1587. doi: 10.3390/v14071587 (PMC9324365; doi:10.3390/v14071587)
Supplement: Supplementary file 1 [file viruses-14-01587-s001.zip › Supplementary Figure S1.pdf]

1-50 window p=0,4997 Recent HIV Chronic HIV

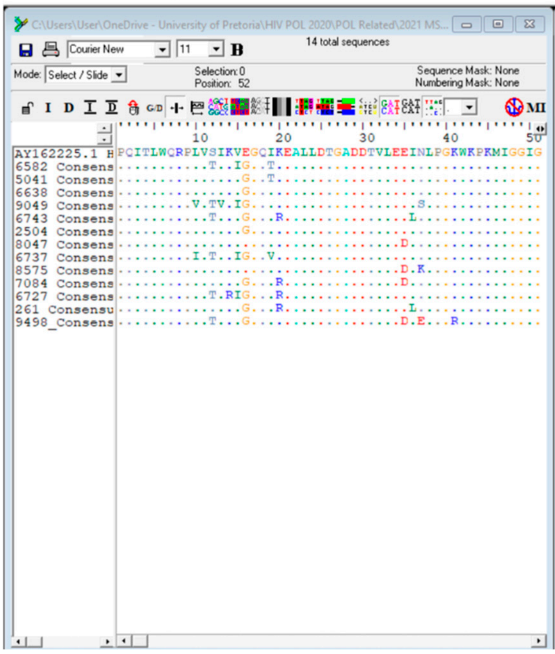

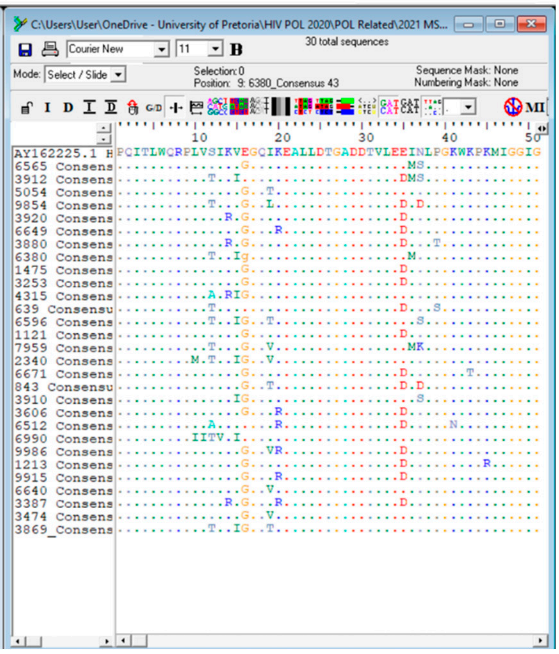

51-100 window p=0,0115 Recent HIV Chronic HIV

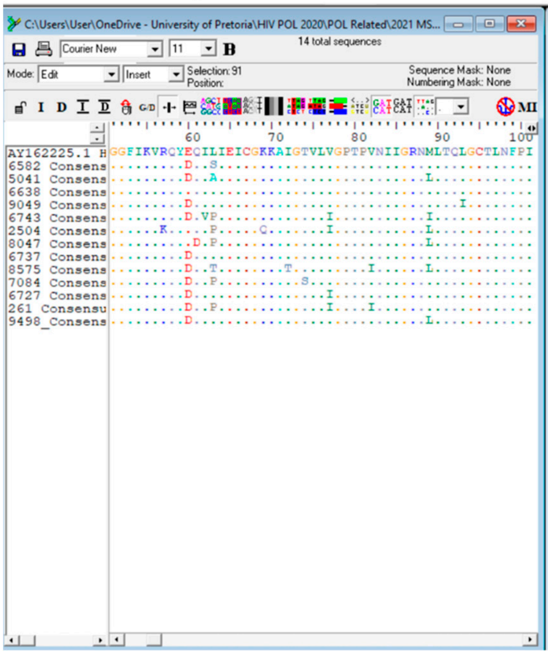

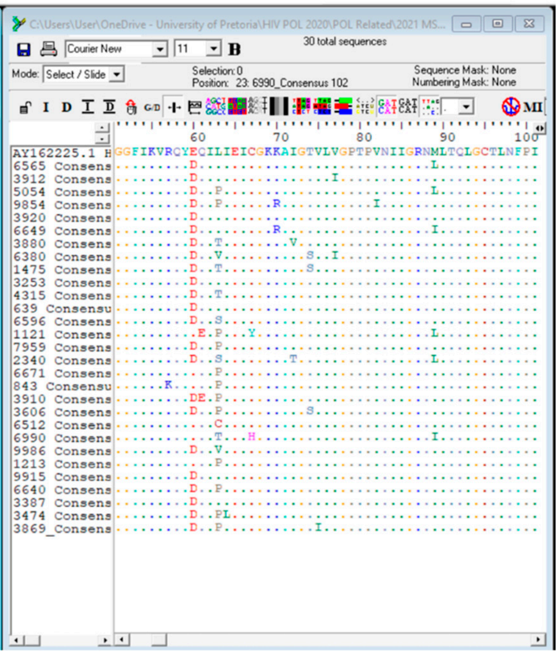

101-150 window p=0,0580 Recent HIV Chronic HIV

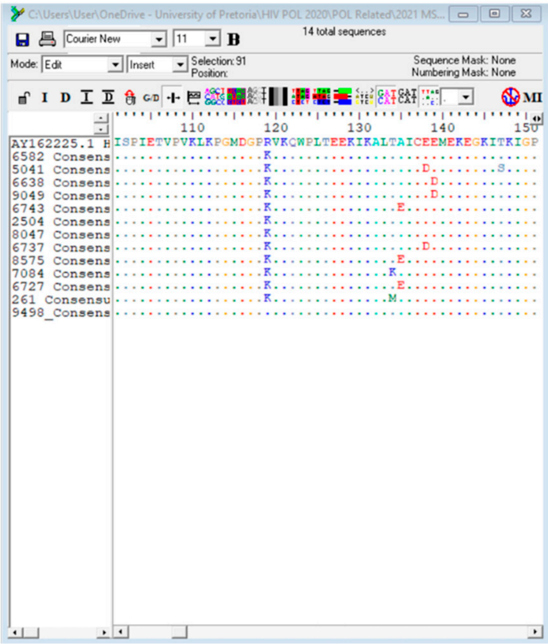

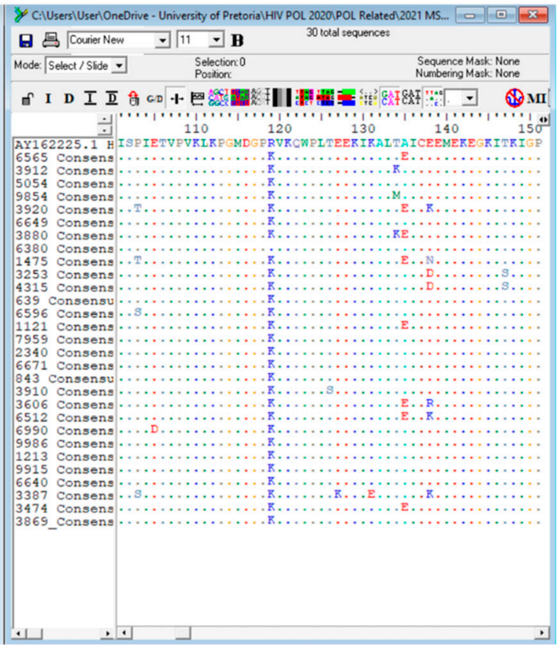

151-200 window p=0,0134 Recent HIV Chronic HIV

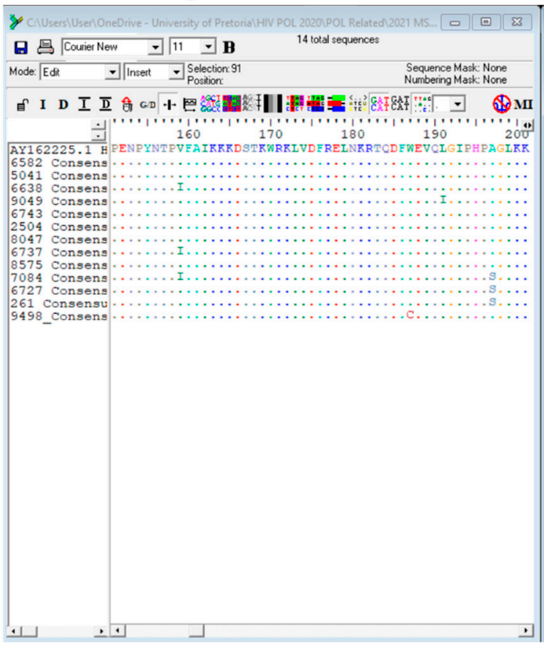

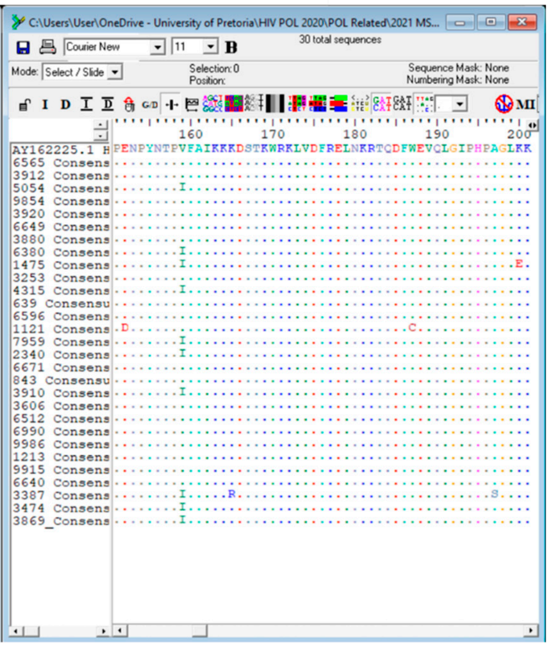

201-250 window p=0,0003

Recent HIV

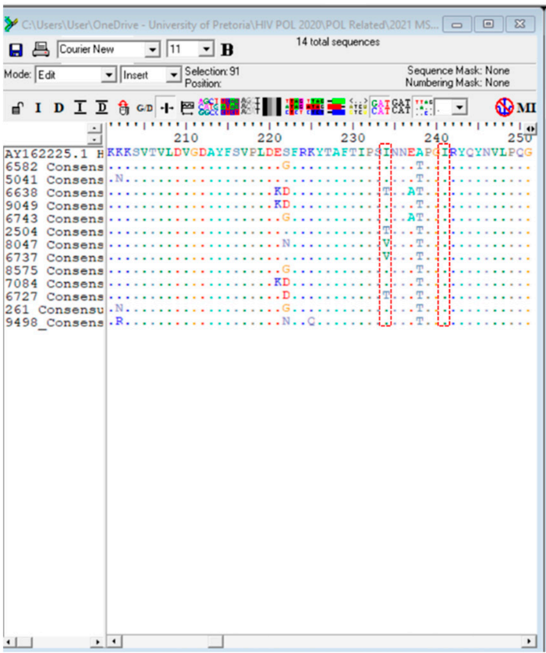

Chronic HIV

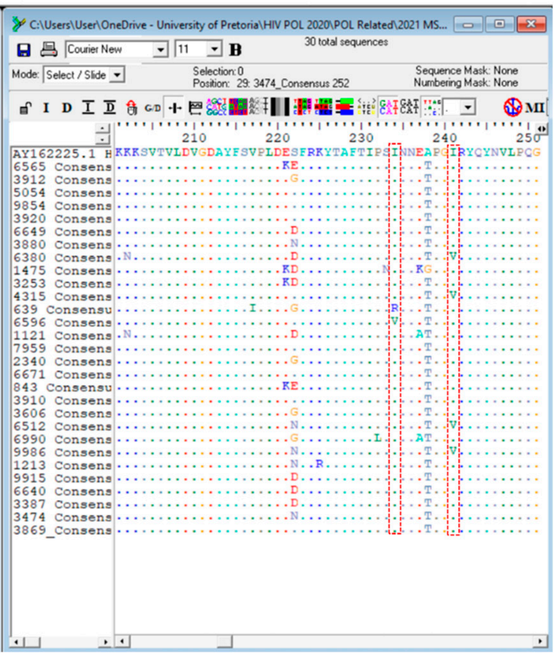

251-300 window p=0,1903

Recent HIV

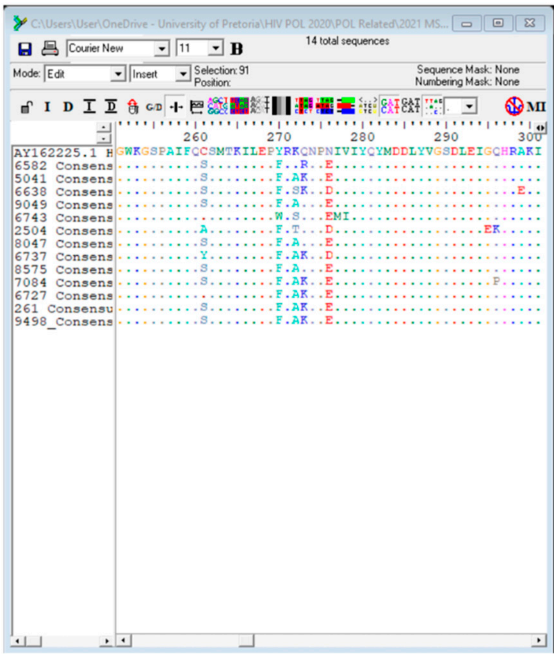

Chronic HIV

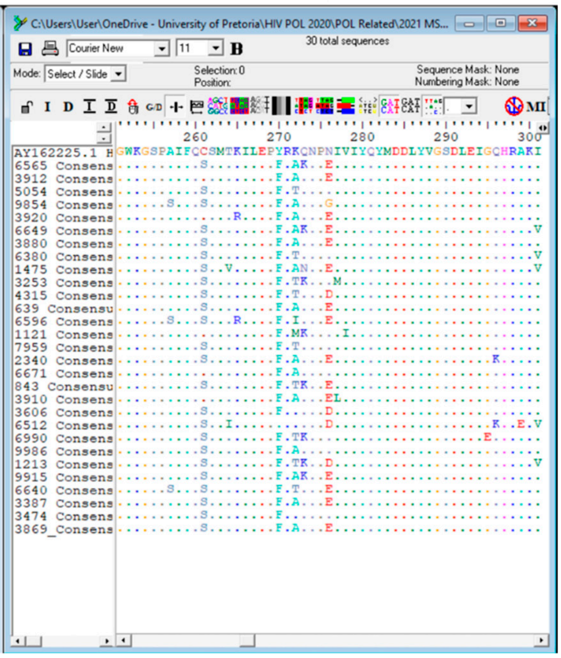

301-350 window p=0,0004

Recent HIV

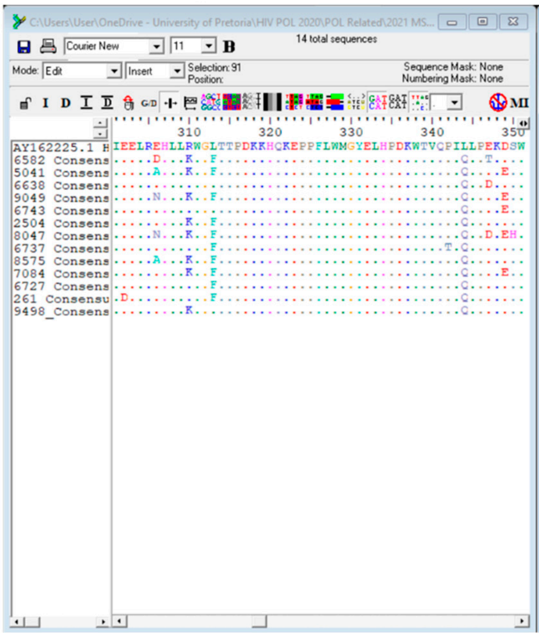

Chronic HIV

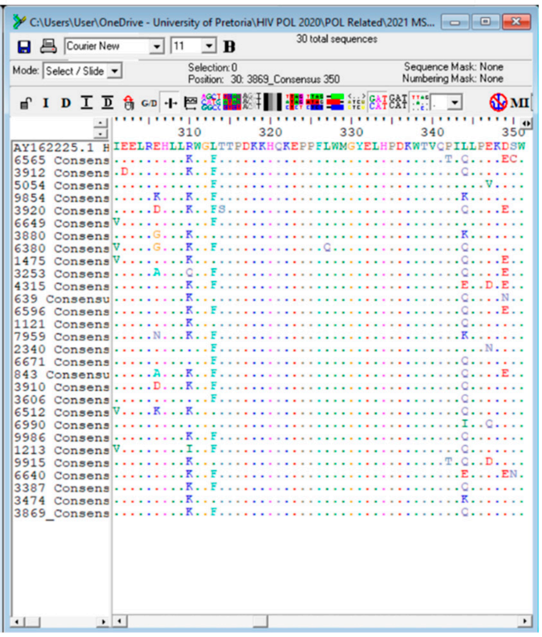

351-400 window p=0,0093

Recent HIV

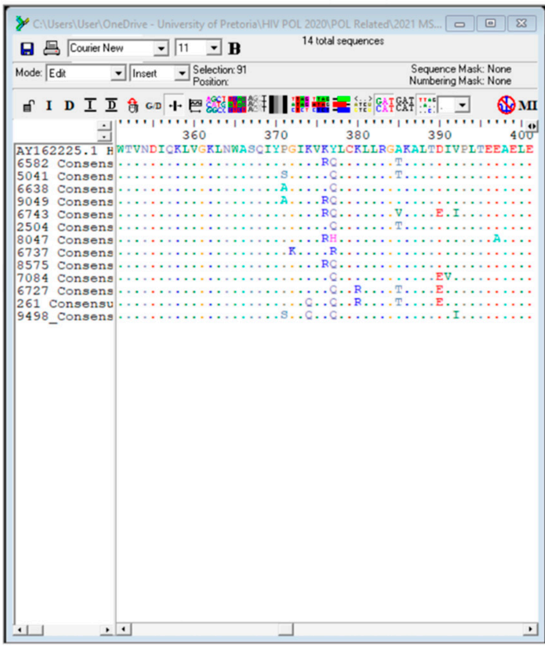

Chronic HIV

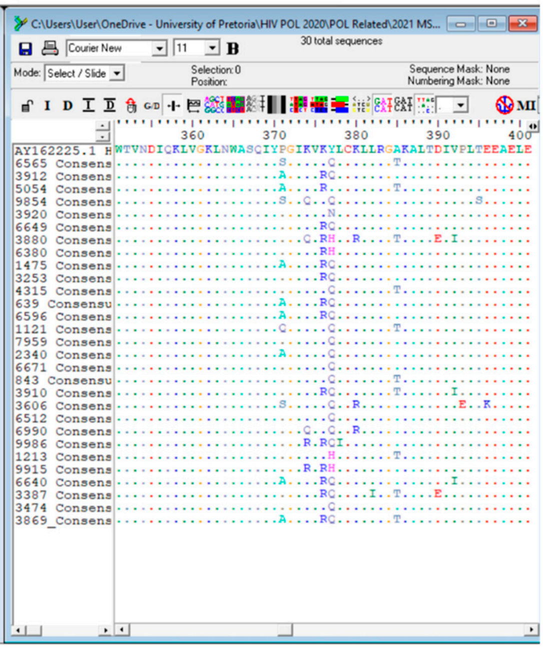

401-450 window p=0,0226 Recent HIV Chronic HIV

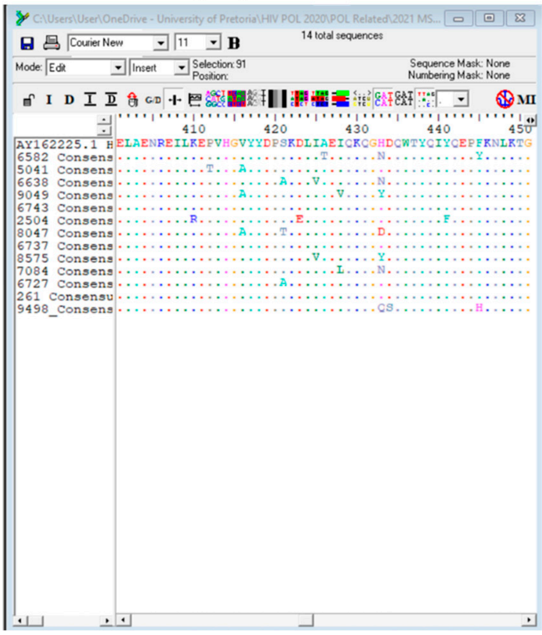

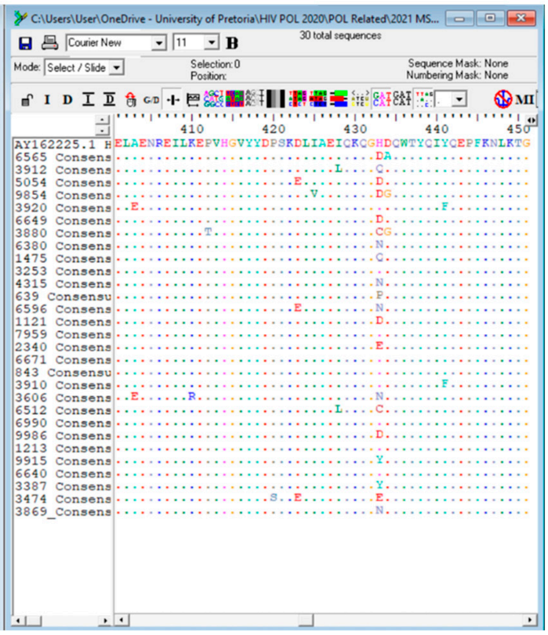

451-500 window p=0,0023 Recent HIV Chronic HIV

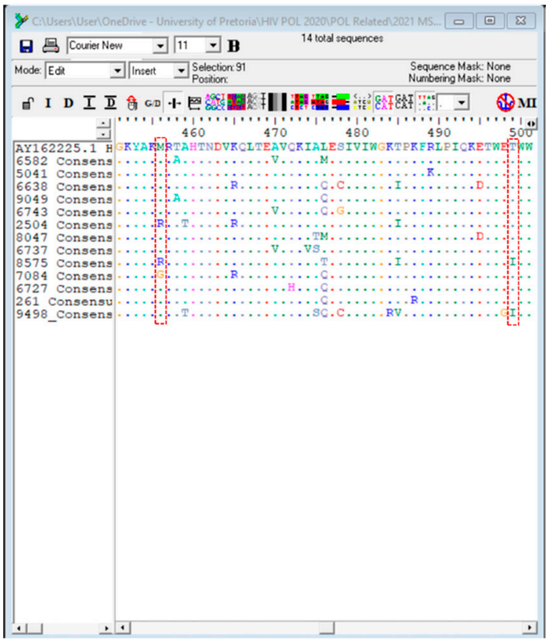

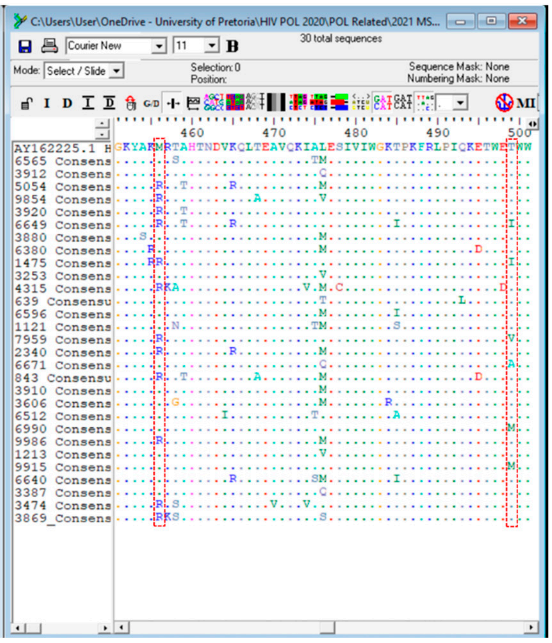

501-550 window p=0,0078 Recent HIV

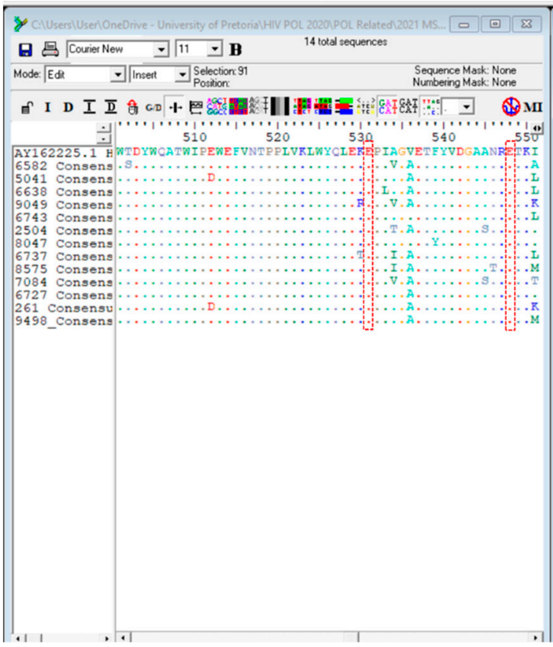

Chronic HIV

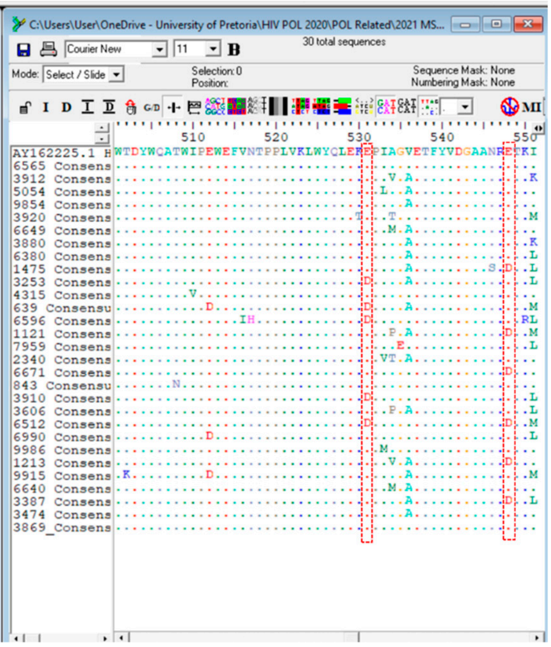

551-600 window p=0,0894 Recent HIV

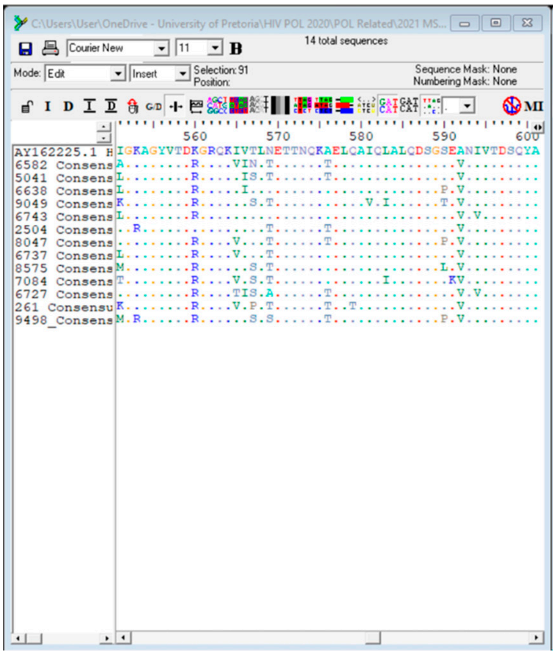

Chronic HIV

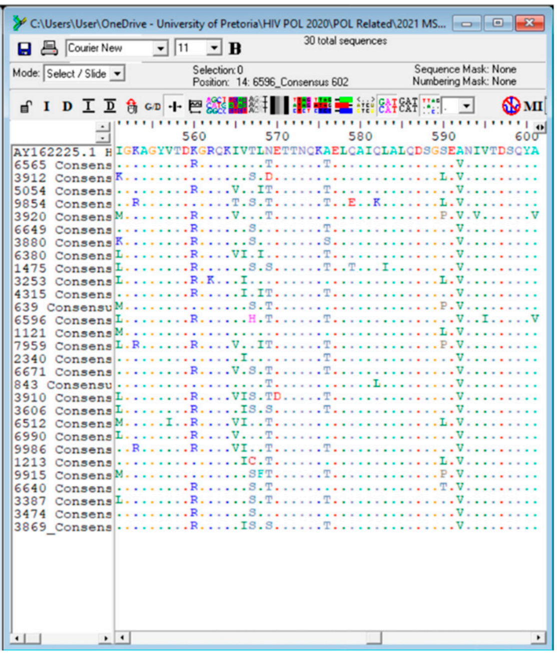

601-650 window p=0,0010 Recent HIV Chronic HIV

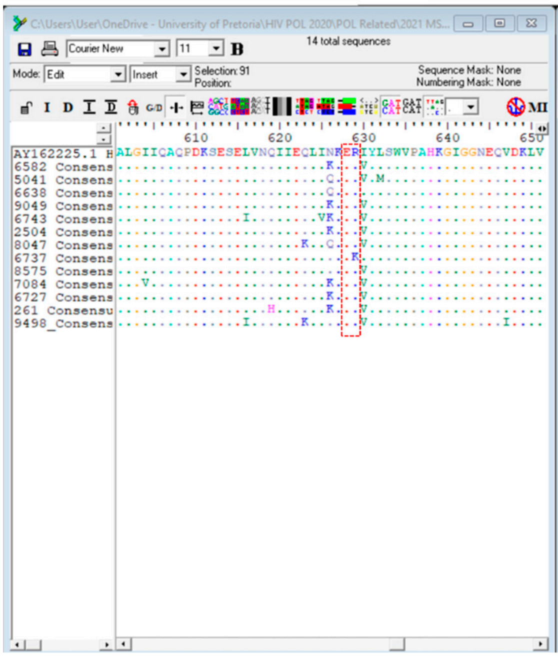

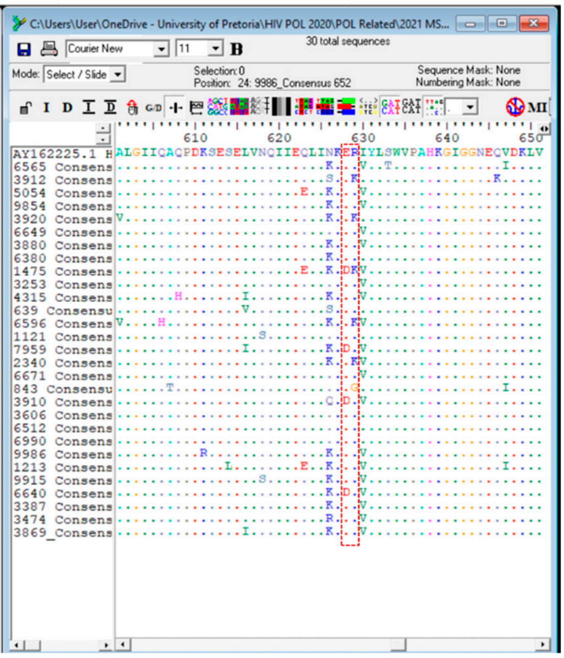

651-700 window p=0,0033 Recent HIV Chronic HIV

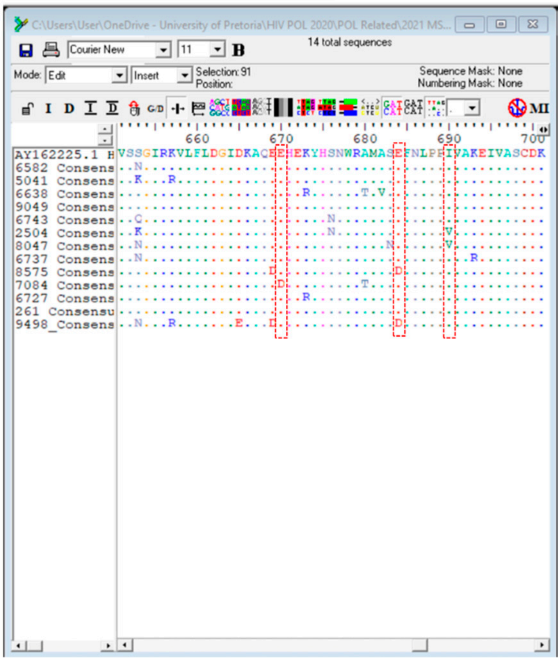

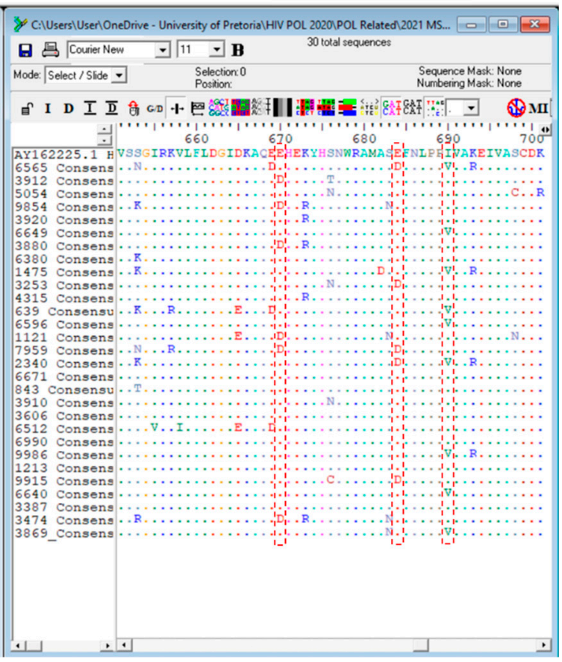

701-750 window p=0,0001 Recent HIV Chronic HIV

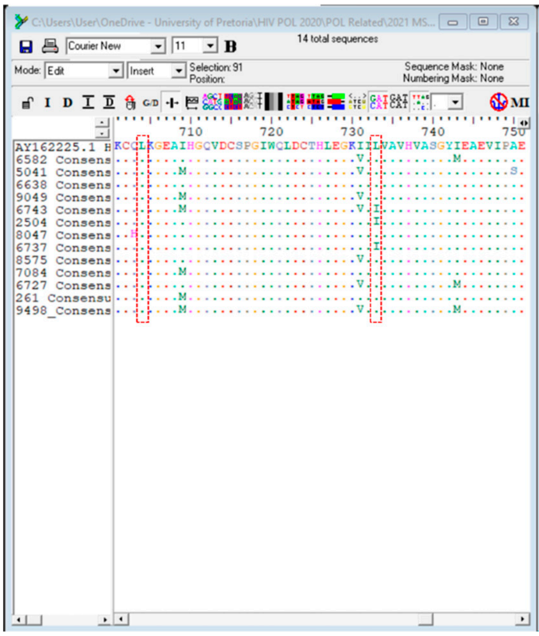

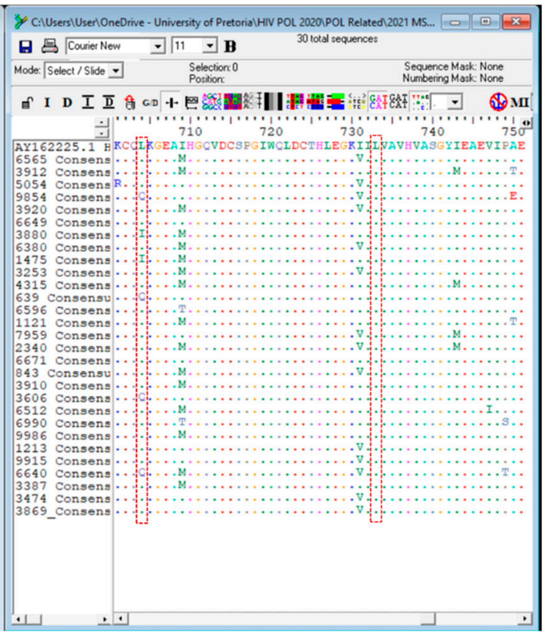

751-800 window p=0,0648 Recent HIV Chronic HIV

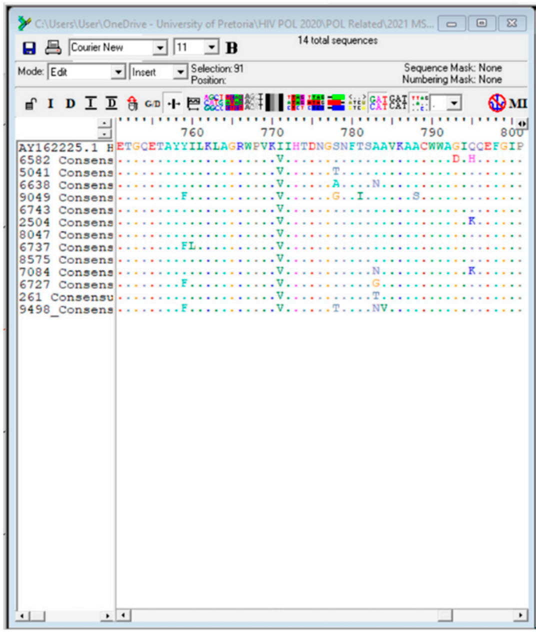

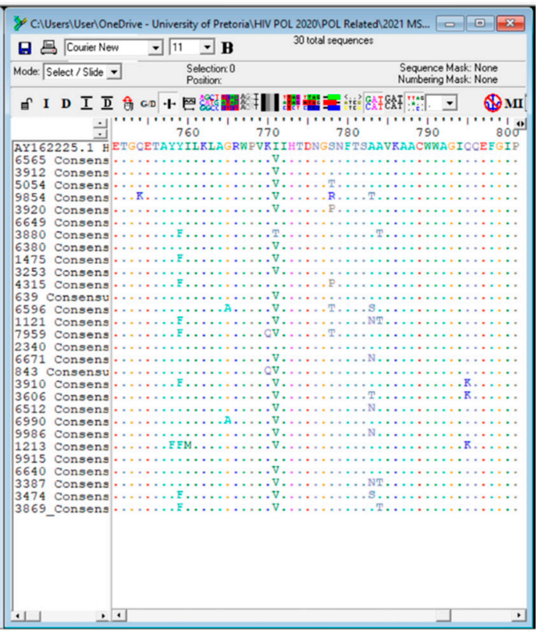

801-850 window p=0,9620 Recent HIV Chronic HIV

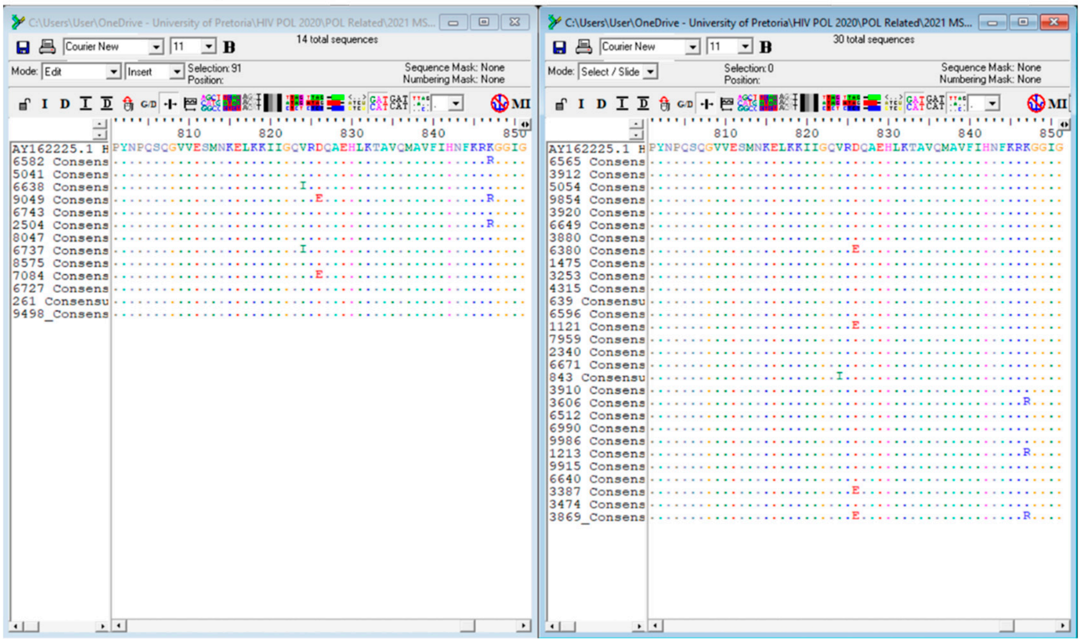

851-900 window p=0,0569 Recent HIV Chronic HIV

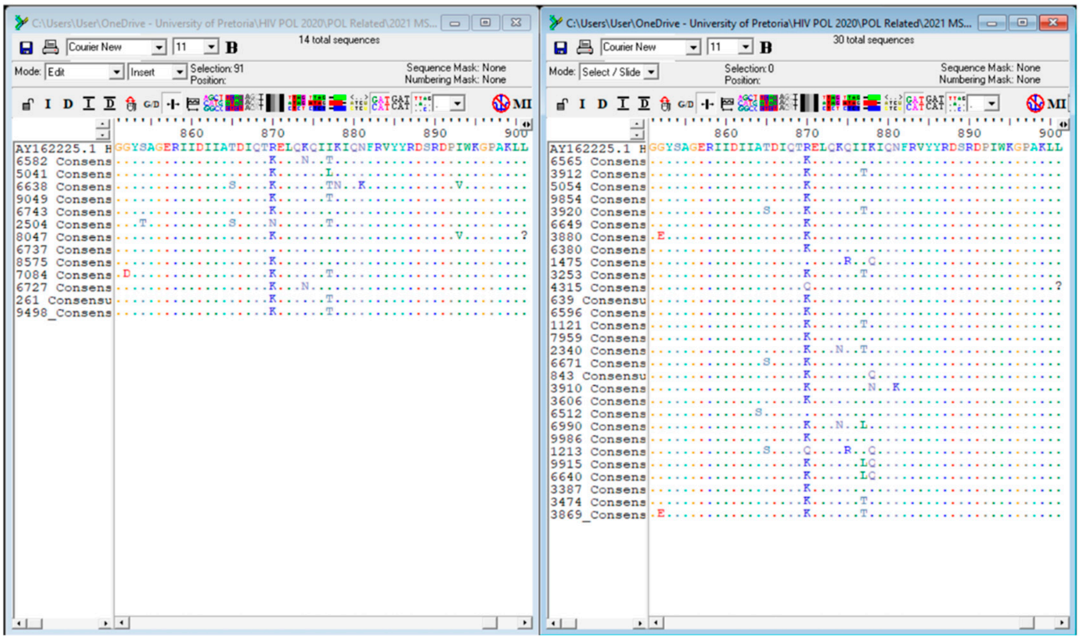

Supplementary Figure S1: Amino acid sequence alignment to show the difference in sequences between recent and chronic HIV-1 infection. The differences were shown for all sliding windows. Positions that showed a significant difference were highlighted in red.
