# Supplementary material for: Evaluation of the HIV-1 Polymerase Gene Sequence Diversity for Prediction of Recent HIV-1 Infections Using Shannon Entropy Analysis
Source: Viruses. 2022 Jul 21;14(7):1587. doi: 10.3390/v14071587 (PMC9324365; doi:10.3390/v14071587)
Supplement: Supplementary file 1 [file viruses-14-01587-s001.zip › Supplementary Figure S2.pdf]

E628 and R629 – Study sequences Recent HIV Chronic HIV

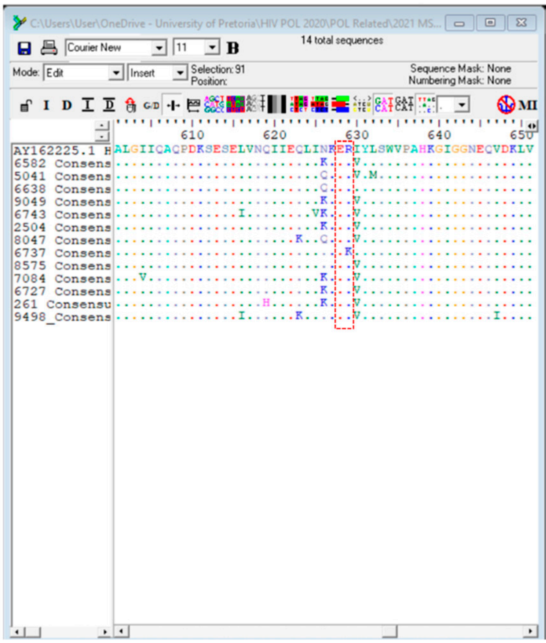

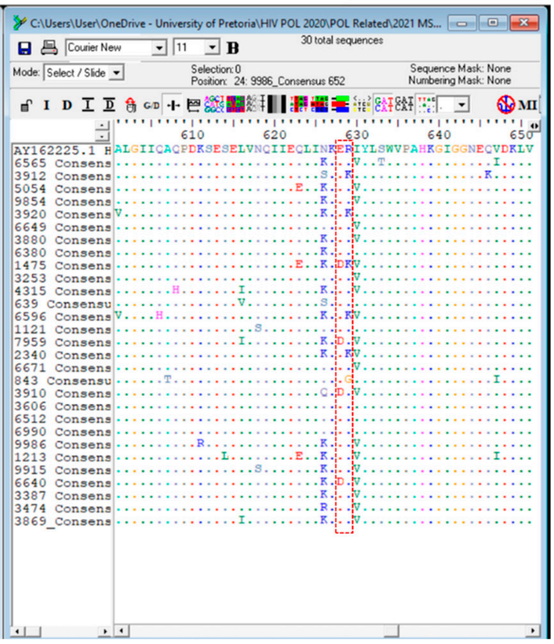

E628 and R629 – Subtype A sequences Recent HIV Chronic HIV

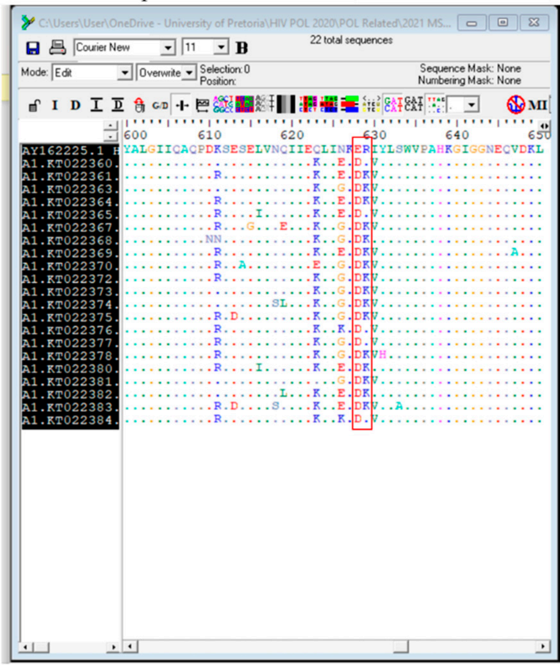

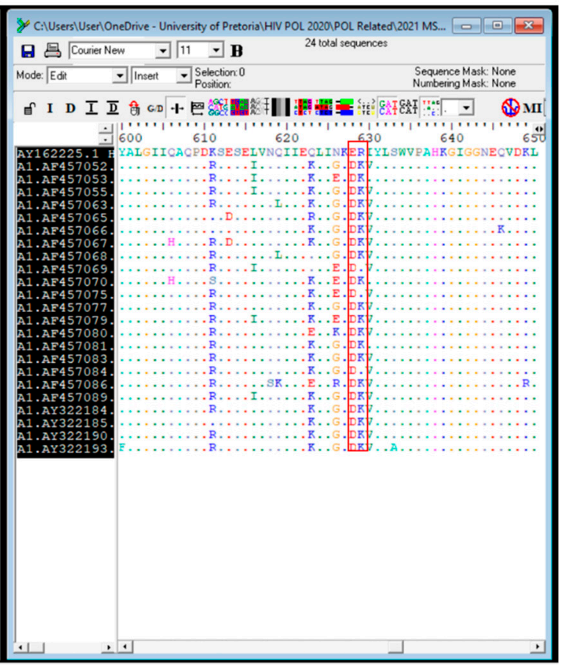

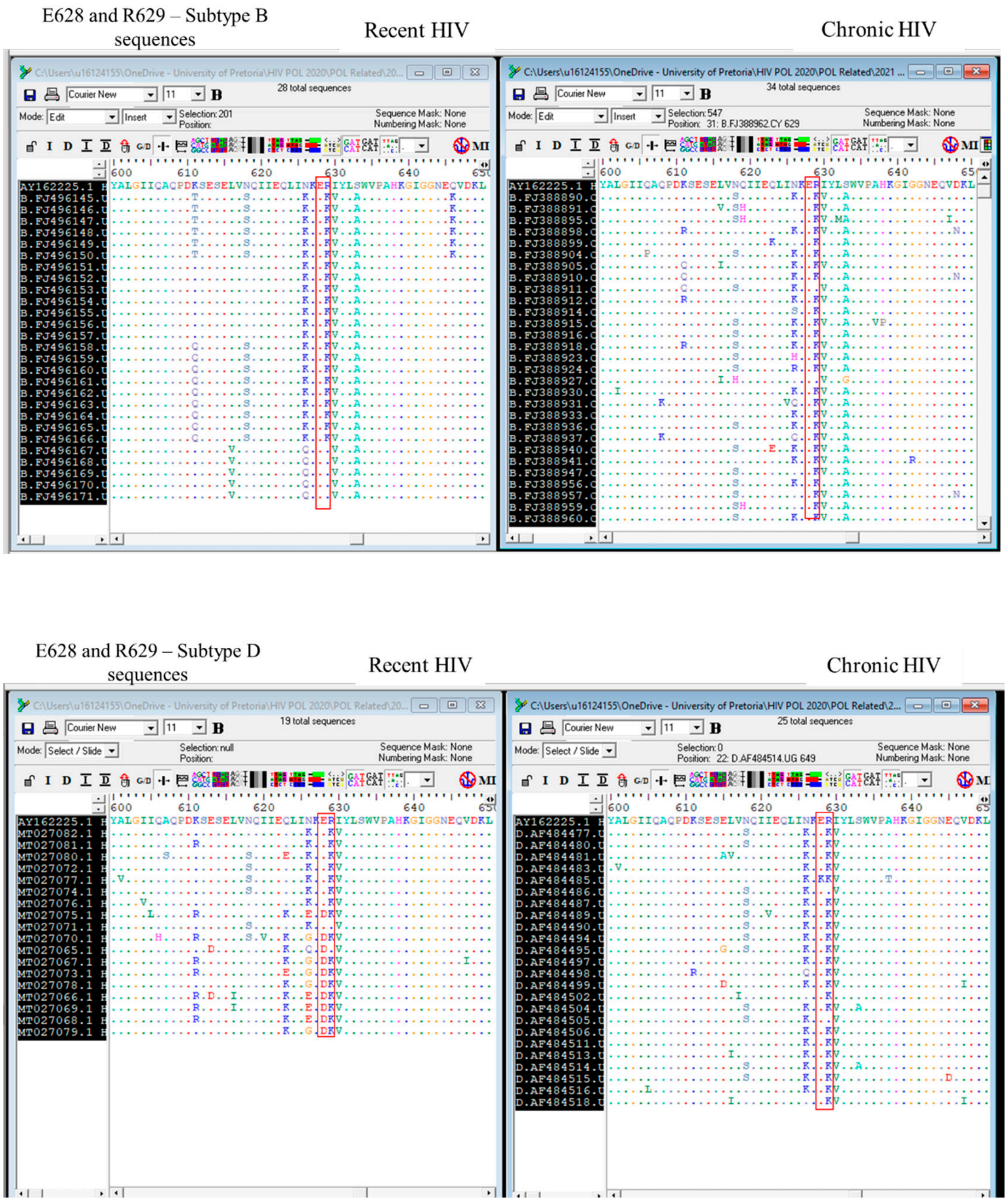

Supplementary Figure S2: Comparison of differences in amino acids E628 and R629 in study sequences and non-subtype C reference sequences, for recent and chronic sequences. For some of the non-subtype C references, the amino acid mutations were found to belong to the wild type of the reference sequence.
