# Supplementary material for: Evaluation of the HIV-1 Polymerase Gene Sequence Diversity for Prediction of Recent HIV-1 Infections Using Shannon Entropy Analysis
Source: Viruses. 2022 Jul 21;14(7):1587. doi: 10.3390/v14071587 (PMC9324365; doi:10.3390/v14071587)
Supplement: Supplementary file 1 [file viruses-14-01587-s001.zip › Supplementary Table S1.pdf]

Supplementary Table S1: List of articles from which HIV-1 subtype C reference sequences were obtained

**Authors**	**Year**	**Fiebig staging**	**Method used to diagnose stage of infection**	**Enzyme used for amplification**	**Country**	**Accession numbers**
Recent HIV articles						
Treurnicht *et al*	2010	I - IV	RNA + p24 + EIA + WB	Expand high fidelity polymerase	South Africa	GQ999972 – GQ999991
Manak *et al*	2012	I - IV	RNA + p24 + EIA + WB	Platinum Taq DNA polymerase	South Africa	JN687620 – JN687651
Parrish *et al*	2012	I - III	RNA + p24 + EIA + WB	Platinum Taq DNA polymerase	Malawi	KC149260 – KC149450
Sanchez *et al*	2014	IV - V	RNA + p24 + EIA + WB	Platinum Taq DNA polymerase	India	KP109480 – KP109489; KX069219 – KX069228
*Song *et al*	2018	Unknown	Unclear	Platinum Taq DNA polymerase	South Africa, Malawi	MF501044 – MF501081
Chronic HIV articles						
Matthews *et al*	2008	N/A	Days post seroconversion	Unknown	South Africa	FJ199915 – FJ199992
Parrish *et al*	2012	N/A	N/A	Platinum Taq DNA polymerase	Malawi	KC156299 – KC156499
Novitsky	1999	N/A	Days post seroconversion	Expand high fidelity polymerase	Botswana	AF110959 – AF110981
*Ojwach *et al*	2018	N/A	Days post infection	TaKaRa Ex Taq polymerase	South Africa, Botswana	MH487430 – MH487469

Song *et al* sequences had a lot of ambiguous bases and were not used for analysis

Ojwach *et al* sequences were only for protease and reverse transcriptase, they were also not used for analysis
