# Supplementary material for: Evaluation of the HIV-1 Polymerase Gene Sequence Diversity for Prediction of Recent HIV-1 Infections Using Shannon Entropy Analysis
Source: Viruses. 2022 Jul 21;14(7):1587. doi: 10.3390/v14071587 (PMC9324365; doi:10.3390/v14071587)
Supplement: Supplementary file 1 [file viruses-14-01587-s001.zip › Supplementary Table S2.pdf]

Supplementary Table S2: Nucleotide differences within amino acid mutations found to have a different distribution between recent and chronic infection sequences

		IT			ER				EL			
Sample	HIV Stage	I240	M456	T499	E531	E548	E628	R629	E670	E684	I690	L704
Codon sequence		ATT	ATG	ACA	GAA	GAA	GAG	AGA	GAG	GAG	ATA	CTA
261	R							--G				
2504	R		-G-				--A				G--	
5041	R						--A					
6512	R						--A					
6727	R						--A	--G				
6638	R						--A	--G	--T			
6582	R						--A					
9498	R						--A	--G		--T		--G
9049	R			-T-			--A	--G				
8575	R	--C	-G-	-T-			--A	--G		--T		
8047	R						--A	--G		--A	G--	
7084	R		GG-				--A	--G	--T	--A		
6743	R						--A	--G				
6737	R						--A	-A-		--A		
639	Chr				--C		--A					-AG
843	Chr		-GA			--T	--A	G--				
1121	Chr			--G	--G	--C	--A		--T	--A		
1213	Chr					--T		--G				
1475	Chr		-G-	-T-		--T	--C	-AG		--A		A--
2340	Chr						--A	-AG		--T		
2696	Chr						--A					
3253	Chr				--C		--A	-AG	--A	--C		
3387	Chr					--C	--A	--G	--A			T--
3474	Chr		-G-				--A	--G	--T			
9986	Chr	G--	-G-				--A	--G				

3606	Chr	--C				--G	--A					-A-
3869	Chr		-G-				--A	--G				
3880	Chr						--A	--G	--C	--A		A--
3910	Chr				--C		--C	-AG	--A			
3912	Chr					--G	--A	-A-	--C			
3920	Chr		-G-				--A	-AG	--A			
4351	Chr	G--	-G-				--A	--G				
4198	Chr						--A					
5054	Chr		-GA				--A	--G				
6380	Chr	G--					--A					T--
6565	Chr						--A			--T		
6671	Chr			G-G		--C	--C	-AG		--T		
6649	Chr		-G-	-T-			--A	--G				
6640	Chr			--T	--C		--T	-AG		--A		-A-
6596	Chr						--A	-AG		--A		
9915	Chr						--A	--G		--T		--G
9895	Chr						--A	--G				
9854	Chr		-G-				--A		--T			-A-
7959	Chr	G--	-G-	GT-	--G		--A	--G	--T	--T		
6990	Chr			-TG			--A	--G				T--

Different groups of amino acid mutations (ER, EL and IT) were named based on the first and last amino acid of the group. HIV = Human immunodeficiency virus; R = recent HIV infection; Chr = chronic HIV infection; A = adenine; G = guanine; T = thymine; C = cytosine
